# Intramuscular Anatomy Drives Collagen Content Variation Within and Between Muscles

**DOI:** 10.3389/fphys.2020.00293

**Published:** 2020-04-17

**Authors:** Benjamin I. Binder-Markey, Nicole M. Broda, Richard L. Lieber

**Affiliations:** ^1^Shirley Ryan AbilityLab, Chicago, IL, United States; ^2^Department of Physical Medicine and Rehabilitation, Northwestern University, Chicago, IL, United States; ^3^Department of Biomedical Engineering, Northwestern University, Chicago, IL, United States; ^4^Edward G. Hines VA Medical Center, Maywood, IL, United States

**Keywords:** skeletal muscle, collagen, connective tissue, anatomy, hydroxyproline, histological analysis

## Abstract

The passive load bearing properties of muscle are poorly understood partly due to challenges in identifying the connective tissue structures that bear loads. Prior attempts to correlate passive mechanical properties with collagen content (often expressed as a mass ratio and used as a surrogate for connective tissue quantity within muscle) have not been successful. This is likely a result of not accounting for variability in intramuscular connective tissue throughout a muscle such that a single collagen content value likely does not adequately represent the connective tissue load bearing capacity of a muscle. Therefore, the purpose of this study was to determine how intramuscular connective tissue distribution throughout a muscle impacts measured collagen content. For this analysis, four mouse hindlimb muscles were chosen because of their varying actions and anatomy; rectus femoris, semimembranosus, tibialis anterior, and lateral gastrocnemius. Collagen content throughout each muscle was determined biochemically using an optimized hydroxyproline assay. Dense connective tissue distribution throughout each muscle’s length was quantified histologically. We found that collagen content varied widely within and between muscles, from 3.6 ± 0.40 SEM μg/mg wet weight to 15.6 ± 1.58 SEM μg/mg, which is dependent on both the specific location within a muscle and particular muscle studied. Both collagen content and connective tissue structures demonstrated stereotypically patterns with the highest quantity at the proximal and distal ends of the muscles. Additionally, using three independent approaches: (1) linear regression, (2) predictive modeling, and (3) non-linear optimization, we found complementary and corroborating evidence suggesting a causal relationship between a muscle’s connective tissue distribution and collagen content. Specifically, we found that muscle collagen content is driven primarily by its dense connective tissue structures due to the extremely high collagen content of connective tissue (227.52–334.69 μg/mg) compared to muscle tissue (1.93–4.03 μg/mg). A consequence of these findings is that a single collagen content measurement does not accurately represent a muscle’s complex distribution of connective tissue. Future studies should account for collagen content variations and connective tissue anatomy to establish more accurate relationships between collagen content measurements and whole muscle passive mechanics.

## Introduction

Skeletal muscle is a heterogeneous composite tissue composed of relatively compliant muscle fibers embedded in a relatively stiff connective tissue matrix. While the major role of muscle is to generate force and movement from muscle fibers, connective tissue is critical to transmit this force as well as to bear active and passive loads throughout the muscle (Purslow, 1989; Gillies and Lieber, 2011). This passive load bearing property is poorly understood (Herbert and Gandevia, 2019) partly because the vast majority of previous research focused on muscle active force production (Lieber and Ward, 2011; Herzog, 2017; Lieber et al., 2017).

Part of the challenge to understanding passive load bearing properties in muscle is properly identifying and quantifying the structures responsible for bearing those loads. As mentioned above, connective tissue is thought to be the primary load bearing structure, but prior attempts to correlate passive mechanical properties with a singular collagen content (often expressed as a mass ratio and used as a surrogate for connective tissue quantity within muscle) have not been successful (Smith et al., 2011; Lieber and Ward, 2013; Chapman et al., 2014; Smith and Barton, 2014). Poor correlations may be the result of not considering the complex distribution of stiff collagen-rich connective tissue structures (internal tendon, aponeurosis, perimysium, endomysium, etc.) within the muscle. One can imagine that, as these stiff collagen-rich structures vary throughout and among muscles, variations would affect a muscle’s passive load bearing capacity while also causing collagen content to vary throughout the muscle. Thus, it may not be surprising that a single collagen value, as used in most previous reports, does not accurately predict the muscle’s passive load bearing capacity.

Unfortunately, there has been no explicit comparison between muscle collagen content and intramuscular connective tissue anatomy that would test this assumption. Therefore, the purpose of this study was to determine how intramuscular connective tissue distribution impacts collagen content measured within muscle. By understanding this relationship, we can establish methods that identify the structures contributing to a muscle’s passive mechanical properties.

## Materials and Methods

### Materials

All procedures within this study were performed in accordance with the NIH Guide for the Use and Care of Laboratory Animals and approved by the Northwestern University’s Institutional Animal Care and Use Committee. This study was conducted using fourteen 12-week-old male and female C57Bl6 mice (Jackson Laboratories, Bar Harbor, ME, United States). Mice were euthanized and the rectus femoris (RF), semimembranosus (SM), tibialis anterior (TA), and lateral gastrocnemius (LG) muscles were immediately dissected. These muscles were chosen because of their varying actions and anatomy (Burkholder et al., 1994; Charles et al., 2016). After dissection, external tendons were removed and muscles were either placed individually in 1.5 mL microcentrifuge tubes and frozen at −80°C for biochemical analysis or pinned to cork and snap frozen in isopentane chilled by liquid nitrogen (−159°C) for histological sectioning. All samples were then stored at −80°C until used.

### Methods

#### Biochemical Collagen Content Quantification

##### Sample preparation

To measure collagen content (defined as a mass fraction, i.e., μg collagen per mg wet muscle mass) variation, 24 muscles (4 muscles × 6 legs) were thawed and, using a scalpel, ∼10 mg blocks were cut from three regions within each muscle. Blocked sections were collected from the most proximal, most distal, and middle regions of the muscle.

To measure the collagen content of the structures likely contributing to the total collagen content throughout muscle, we divided the tissue into two categories: (1) baseline muscle (muscle tissue with its associated endomysium and perimysium but no large dense connective tissue structures) and (2) dense connective tissue (internal tendons and aponeuroses). The process for separating these tissues is described below. Collagen content of each tissue was measured separately.

To measure baseline muscle collagen content, 24 muscles (4 muscles × 6 legs) were thawed and ∼10 mg samples from the mid belly of each muscle were collected. Dense connective tissues such as distinct internal tendons of the TA and RF and aponeuroses of the RF and LG were removed from the sample by manually microdissecting the muscle tissue from the connective tissues.

To measure the dense connective tissue structures’ collagen content; 18 muscles [three muscles (RF, TA, and LG) × 6 legs) were allowed to thaw, internal tendons from RF and TA and aponeurosis of the RF and LG were collected by microdissecting the structures from the muscle tissue, leaving only the connective tissue structure (ranging in mass from 0.32 to 1.47 mg).

##### Hydroxyproline colorimetric assay

Collagen content for each sample was determined biochemically using colorimetric hydroxyproline assay, modified from a previously described protocol (Reddy and Enwemeka, 1996). Briefly, the assay was performed on samples placed in 1 mL of 6N HCl in a sealed screw top culture tube and hydrolyzed for 24 h at 110°C. Initial validation tests determined that 24 h was the optimal time for hydrolysis of these samples (validation tests are described below and presented graphically in Supplementary Figures 1, 2). At 24 h, samples were removed, cooled to room temperature, and plated in triplicate. Plated samples were evaporated then treated with a chloramine T solution for 20 min at room temperature followed by a solution of *p*-diaminobenzaldehyde for 30 min at 60°C. Absorbance was read at 550 nm and compared to a standard curve to quantify the hydroxyproline content. Hydroxyproline content was converted to collagen content using the constant (7.46) that defines the number of hydroxyproline residues per collagen molecule.

##### Muscle collagen hydrolysis timing validation

To ensure complete and optimal hydrolyzation of collagen within our muscle tissue samples, hydroxyproline content of the muscle tissue samples described above were hydrolyzed and tracked over 72 h. Additionally, to determine optimal collagen hydrolyzation time, hydroxyproline content of human type I (C5483, Sigma-Aldrich Co., St. Louis, MO, United States) and III collagen (CC054, EMD Millipore Corp., Billenca, MA, United States) at three concentrations (10, 20, and 30 μg/mL) was quantified at specific intervals over 72 h of hydrolysis. As in the protocol described above, samples were hydrolyzed at 110°C in 1000 μL of 6N HCl in a sealed screw top culture tube. At 2, 4, 6, 8, 10, 24, 48, and 72 h, 90 μL aliquots from culture tubes were plated in triplicate and immediately set on ice and refrigerated to halt hydrolysis then processed and analyzed as described above.

#### Histological Quantification of Muscle Dense Connective Tissue Distribution Along Muscle Length

Muscle samples stored at −80°C for histological sectioning were raised to −20°C before transfer to molds with chilled Frozen Section Compound (FSC 3801480, Leica, Buffalo Grove, IL, United States), then quickly snap frozen in isopentane chilled by liquid nitrogen (−159°C). Molds were transferred to a cryostat (CM19050 Leica, Buffalo Grove, IL, United States), brought to −20°C, and mounted in the cryostat to allow transverse 10 μm sections along the entire muscle length.

Sections were fixed for 3 min in 10% glutaraldehyde and rinsed continuously in deionized (DI) water for 1–2 min. Excess water was shaken off and slides were dried at room temperature for 10 min. Once the slides were fully dried, they were incubated for 60 min at room temperature in a dye chamber filled with Picosirius Red (s2365 Poly Scientific, Bay Shore, NY, United States). Sections were then washed twice in 0.1 N HCl for 1 min then rinsed continuously with DI water for 1–2 min. Sections were then subjected to a dehydration sequence: 70, 80, 95, 100, 100% ethanol for 1 min each. Sections were then cleared with xylene for 15–30 min and wet mounted with a xylene mounting medium (Cytoseal-60 8310-4, Thermo Scientific, Waltham, MA, United States) and coverslip.

Images were taken under 10× magnification and stitched together using a preset algorithm on a Leica Upright DM6000 (Leica, Buffalo Grove, IL, United States) system. Stitched images were imported into Photoshop 2018 (Adobe, San Jose, CA, United States) to determine the area fraction per section of dense connective tissue structures; internal tendons, and aponeuroses. The number of pixels corresponding to the connective tissue structures over the total pixel number of the muscle image defined connective tissue area fraction. Pixels corresponding to the connective tissue structures of the internal tendon and aponeurosis were determined within Photoshop using the built in “magic wand” tool that allows the user to manually select an initial seeding point and the tool selects contiguous pixels within a tight color range of the initial seeding point. The user then edits the selected pixels manually.

**FIGURE 1:**
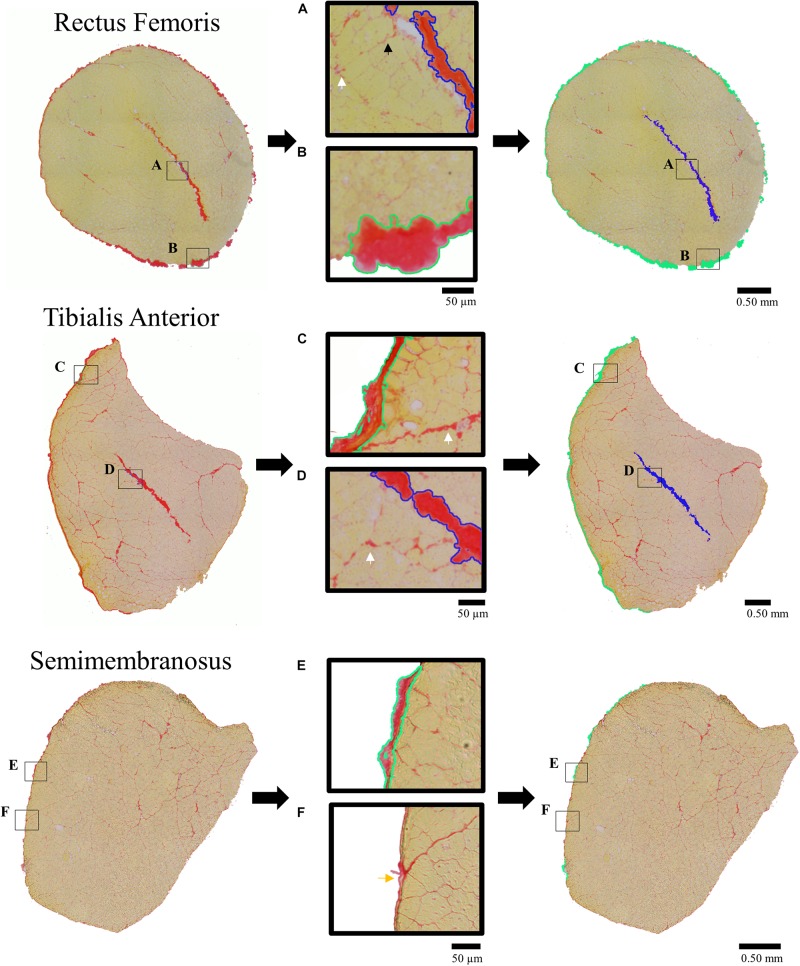
Picrosirus red stained histological sections (left column) demonstrating tracing procedure for quantification of dense connective tissue structures (right column) of the internal tendons (shaded blue) and aponeurosis (shaded green) for the Rectus Femoris (RF), Tibialis Anterior (TA), and Semimembranosus (SM). Middle column presents enlarged portions demonstrating tracing of the connective tissue structures that were included in the area fraction analysis. Callouts illustrate the tracing of the **(A)** RF internal tendon (blue outline), **(B)** RF aponeurosis (green outline), **(C)** TA aponeurosis (green outline), and **(D)** TA internal tendon (blue outline) with endomysium (small black arrow) and perimysium (white arrow) between fascicles surrounding neurovascular tracts that were not quantified as dense connective tissue structures. Discrimination between **(E)** SM sheath included in the analysis (green outline) and **(F)** SM epimysium not included in the analysis (yellow arrow) were based on their relative size, continuity, and shape.

To be consistent with the biochemical analysis, only dense connective tissues (internal tendons and aponeuroses) were quantified while enlarged areas of loose connective tissue structures (perimysium and connective tissue surrounding neurovascular tracts) within the muscle were not quantified as they were considered part of the “baseline” muscle tissue (Figure 1). To confirm this assumption, we quantified loose connective tissues and repeated the analyses conducted in Section “Quantifying the Relationship Between Muscle Dense Connective Tissue and Collagen Content” to include the loose connective tissue structures. This analysis confirmed that the contribution from the loose connective tissue structures to total collagen content was negligible compared to the dense connective tissue and muscle tissue (see Supplementary Material). Internal tendons and aponeuroses were visually discriminated from endomysium, perimysium, and epimysium (see white, black, and yellows arrows in Figure 1) based on relative shape, size and location. Qualitatively, internal tendons and aponeuroses appeared thick and had linear continuity across images that made them easily distinguishable from other connective tissue structures. This process of selection and analysis required approximately 30 min to 1 h per image and was completed by a single skilled rater. This resulted in high intra-rater reliability with an intraclass correlation coefficient of 0.996 and average error of 4.54% which was calculated from three repeat analyses of ten histological sections.

#### Quantifying the Relationship Between Muscle Dense Connective Tissue and Collagen Content

We used three independent approaches to describe the relationship between the dense connective tissue distribution measured histologically as area fraction and collagen content measured biochemically as mass fraction.

In the first approach, simple linear regression was applied to the biochemically measured collagen content and histologically measured area fraction across all muscles and regions. Within this analysis, average collagen content across all muscles and regions was used as the dependent variable. The independent variable was the average summed area fraction from the corresponding histological sections. Summed area fractions within a muscle and region were calculated by summing the areas of connective tissues structures, *A*_ct_, of 4–6 consecutive histological sections, representing the volume of tissue analyzed biochemically, then dividing by the sum of the total muscle area, *A*_M_, across those sections.

(1) $$\sum A\,F_{c\,t}=\frac{\sum_{i=1}^{n}A_{c\,t,i}}{\sum_{i=1}^{n}A_{M,i}},\text{where}\,n=4-6$$

In the second approach, the relationship between collagen content and connective tissue quantity was modeled and compared to the experimentally determined relationship. This modeled relationship was defined by a law of mixtures model that predicts total collagen content of a muscle sample considering the heterogenous composite nature of the tissue.

(2) $${\text{Col}}_{\text{tot},p}=\frac{{\text{Col}}_{c\,t}\,V_{c\,t}\,\rho_{c\,t}+{\text{Col}}_{m}\,V_{m}\,\rho_{m}}{V_{c\,t}\,\rho_{c\,t}+V_{m}\,\rho_{m}}$$

(3) $$V_{m}=1-V_{c\,t}$$

where predicted total collagen content, Col_tot_*_,p_*, was calculated throughout a range of connective tissue volume fractions, *V*_*ct*_, using the biochemically measured collagen content of dense connective tissue, Col*_*ct*_*, and baseline muscle, Col*_*m*_*, from above and tissue densities from the literature, ρ_*ct*_ = 1.12 g/cm^3^ (Ker, 1981) and ρ_*m*_ = 1.06 g/cm^3^ (Mendez and Keys, 1960). If Eq. 2 accurately represents the way in which total collagen content is reflected by its connective tissue area fraction, plotting biochemically measured collagen content across muscle and regions with respect to area fraction (as an approximation of volume fraction) should fall on the relationship predicted by Eq. 2.

The third approach used to understand these relationships was to use optimization to predict Col*_*ct*_* and Col*_*m*_* from biochemically measured tissue collagen content and histologically measured area fractions. Col*_*ct,p*_* and Col*_*m,p*_* were solved via an optimization algorithm minimizing the difference between the measured total collagen content and predicted collagen content using the above law of mixtures model (Eq. 2) across all muscles and regions.

(4) $$\min_{{\text{Col}}_{c\,t,p},{\text{Col}}_{m,p}}\left[{\text{Col}}_{\text{tot}}^{M,r}-\frac{{\text{Col}}_{c\,t,p}\,\sum A\,F_{c\,t}^{M,r}\,\rho_{c\,t}+{\text{Col}}_{m,p}\,\sum A\,F_{m}^{M,r}\,\rho_{m}}{\sum A\,F_{c\,t}^{M,r}\,\rho_{c\,t}+\sum A\,F_{m}^{M,r}\,\rho_{m}}\right]$$

where ${\text{Col}}_{\text{tot}}^{M,r}$ is the average collagen content measured in a specific muscle (*M*) and region (*r*) and $\sum A\,F_{\text{ct}}^{M,r}$ is the average summed area fraction of that respective muscle and region used to represent the volume fraction, *V*_ct_. Optimization was performed in MATLAB (Natick, MA, United States) using the non-linear equation solver *fsolve.*

#### Statistical Analysis

Significance (α) was set to 0.05 for all analyses. To analyze collagen content variations among muscles and regions, a mixed effect model was performed with random factor of animal and fixed factors of muscle and region. To analyze differences among baseline muscle collagen content and dense connective tissue collagen content, mixed effect models were performed using random factor of animal and fixed factor of muscle or structure, respectively. Data are presented in text and figures as average ± SEM unless otherwise noted.

## Results

### Collagen Content Variations Across Muscles and Regions

**FIGURE 2:**
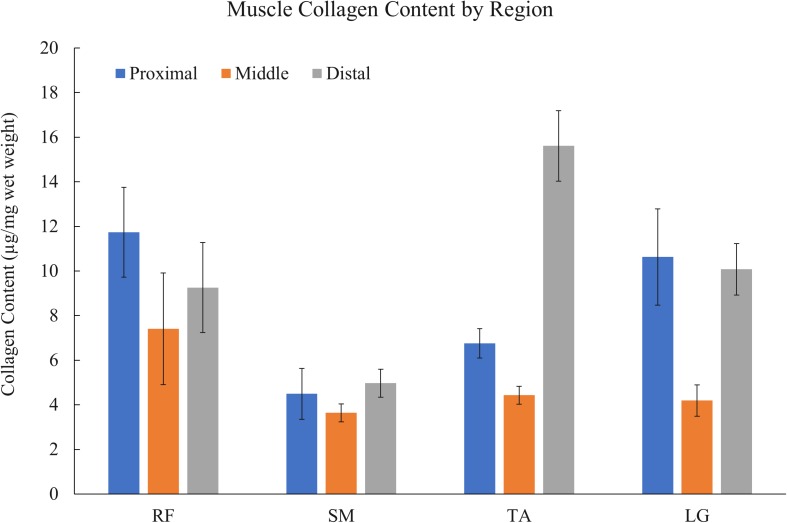
Muscle collagen content variation by region (*n* = 6 muscles/bar) of rectus femoris (RF), semimembranosus (SM), tibialis anterior (TA), and lateral gastrocnemius (LG) muscles. Collagen content varies widely within and between muscles with the stereotypical pattern being higher collagen content at proximal and distal ends. Mixed effect model analysis revealed a significant effect of muscle, region, and muscle × region interaction (all *p* < 0.001) (*n* = 6).

Collagen content varied widely depending on the specific muscle (RF, SM, TA, or LG) and region (proximal, middle, or distal) (Figure 2) from a low of 3.6 ± 0.40 μg/mg wet weight in the middle region of the SM to a high of 15.6 ± 1.58 μg/mg in the distal region of the TA (*n* = 6/muscle). Statistical analysis revealed a significant effect of muscle, region, and muscle × region interaction (all *p* < 0.001) (*n* = 6). All muscles demonstrated a stereotypical collagen content pattern wherein proximal and distal ends had higher collagen content while the middle had the lowest collagen content (Figure 2). Importantly, the significant interaction term explicitly demonstrates that collagen content depends both on the region of a muscle and the particular muscle studied.

### Baseline Muscle and Dense Connective Tissue Collagen Content

**FIGURE 3:**
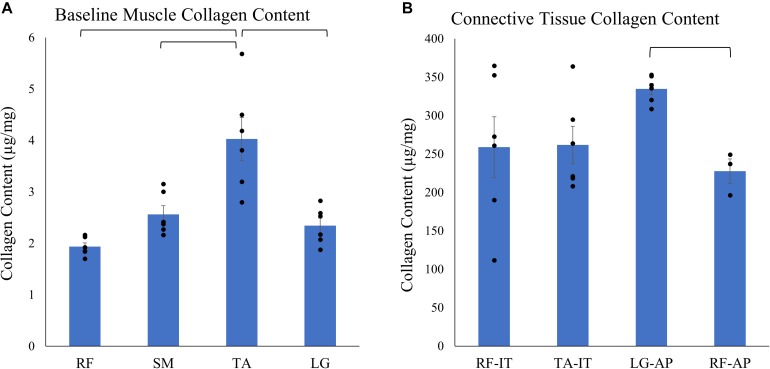
Average ± SEM of **(A)** baseline muscle collagen content (*n* = 6/muscle) of the rectus femoris (RF), semimembranosus (SM), tibialis anterior (TA), and lateral gastrocnemius (LG) and **(B)** connective tissue structures collagen content of the internal tendons of the rectus femoris (RF-IT) and tibialis anterior (TA-IT; *n* = 6/structures) and aponeurosis of the lateral gastrocnemius (LG-AP; *n* = 6) and rectus femoris (RF-AP; *n* = 3). Note that collagen content in connective tissue structures is almost 100 times that of pure muscle tissue. Black points represent the individual data points. Mixed effect models demonstrate that there is a significant effect of muscle on baseline muscle collagen content but there is no effect of the dense connective tissue structure on collagen content. *Post hoc* pairwise comparisons demonstrate significant differences between muscles and structures as denoted by brackets.

Baseline muscle (muscle tissue with its associated endomysium and perimysium but no large connective tissue structures) collagen content across muscles was two orders of magnitude smaller compared to collagen content of dense connective tissue (internal tendons and aponeuroses) (Figure 3). The baseline muscle collagen content ranged from 1.93 ± 0.07 μg/mg wet weight in the RF to 4.03 ± 0.42 μg/mg in the TA (Supplementary Table 1). In contrast, collagen content of connective tissue structures ranged from a low of 227.52 ± 15.99 μg/mg in the RF aponeurosis to a high of 334.69 ± 7.14 μg/mg in the LG aponeurosis (Supplementary Table 2). Analyzing the effect of muscle on the baseline muscle collagen content a mixed effect model revealed a significant effect of muscle (*n* = 6/muscle, *p* < 0.001). However, *post hoc* pairwise comparisons demonstrated only the collagen content of the TA as significantly different than the other muscles (Figure 3A). Conversely, collagen content did not vary significantly (*p* = 0.09) among connective tissue structures (*n* = 6/structure, except RF aponeurosis *n* = 3).

### Histologically Quantified Muscle Connective Tissue Distribution Along Muscle Length

**FIGURE 4:**
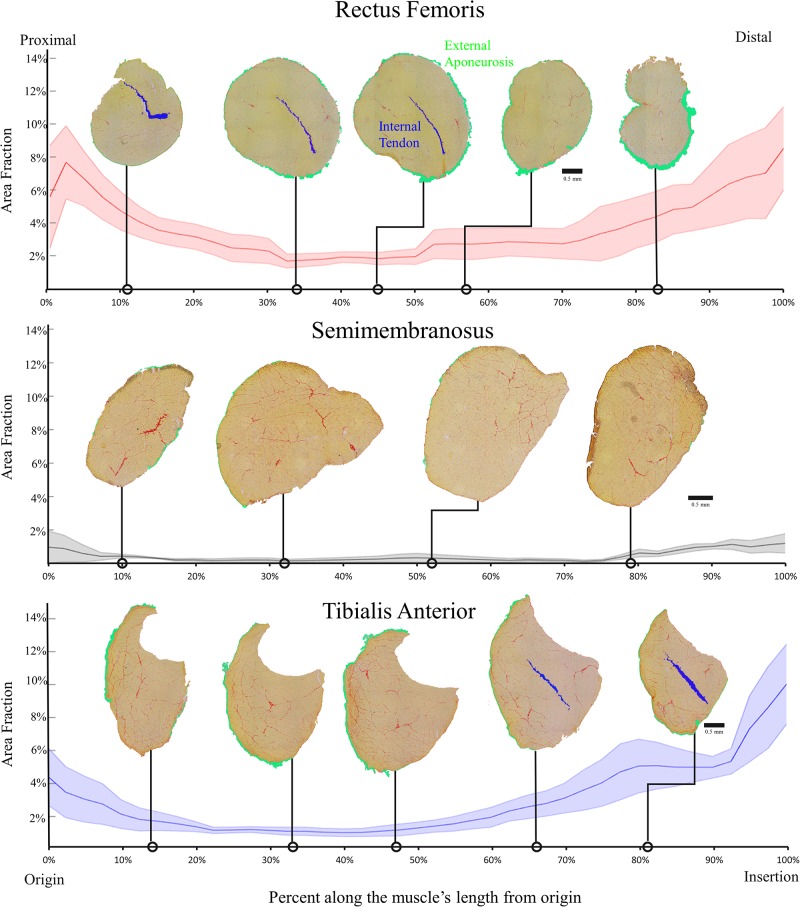
Average area fraction ± SEM (shaded region) along the normalized length of the rectus femoris **(top)**, semimembranosus **(middle)**, and tibialis anterior **(bottom)** (*n* = 4/muscle). The area fraction of the rectus femoris and tibialis anterior demonstrate the pattern of higher area fraction at the proximal and distal ends with low area fraction in the middle. The semimembranosus demonstrates the same pattern but to a much smaller extent since there are minimal connective tissue structures within that muscle. Structures are color coded as shown in the top panel.

Each muscle demonstrated a stereotypical anatomical connective tissue pattern with the greatest area fraction of connective tissue at the ends of each muscle (Figure 4). This stereotypical pattern mimicked the pattern of higher collagen content measured biochemically in the proximal and distal regions of each muscle, suggesting a relationship between connective tissue structures and collagen content (compare Figures 2 and 4).

### Relationship Between Muscle Connective Tissue Distribution and Collagen Content

To understand and quantify the relationship between muscle connective tissue distribution measured histologically and collagen content measured biochemically three independent approaches were taken as described in the section “Methods”: (1) Linear regression, (2) Predictive modeling, and (3) Non-linear optimization. The three independent approaches provided complementary and corroborating evidence of a strongly correlated relationship between muscle connective tissue quantity and collagen content.

**FIGURE 5:**
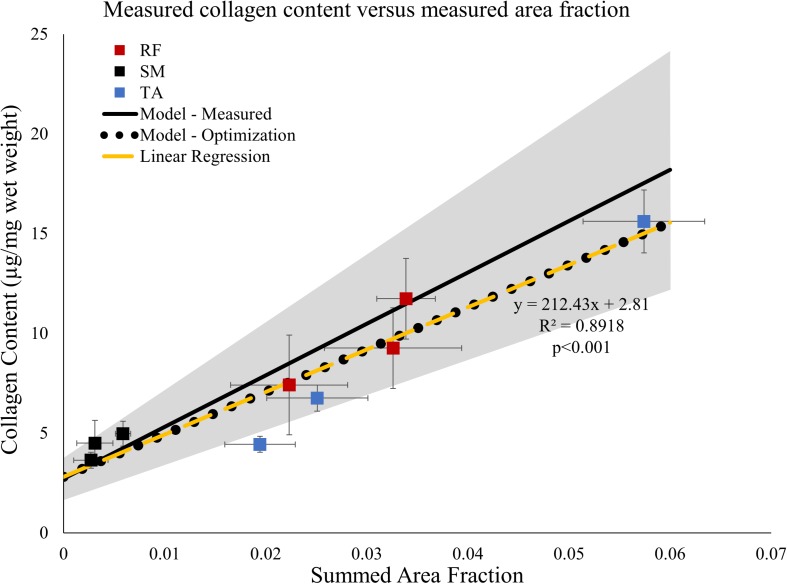
Average measured collagen content ± SEM (*n* = 6/point) from data in Figure 1 versus area fraction ± SEM (*n* = 4/point) from the proximal, middle, and distal regions of the rectus femoris (RF), semimembranosus (SM), and tibialis anterior (TA). Linear regression (dashed yellow line) demonstrates the data are strongly correlated. Solid black line represents predicted relationship based on the law of mixtures (Eq. 2). The gray shaded region represents ± one standard deviation of the measured connective tissue and baseline muscle collagen content. Values used for the law of mixtures model: Col*_*ct*_* = 276.85 μg/mg wet weight, Error bars not needed since a constant value is used in the simulation. ρ*_*ct*_* = 1.12 mg/mm^3^, Col*_*m*_* = 2.72 μg/mg, and ρ*_*m*_* = 1.06 mg/mm^3^. The relationship between collagen content and area fraction using the optimized predicted values of connective tissue and muscle (black dotted line) lies along the relationship predicted by the linear regression (dashed yellow line).

Linear regression of collagen content versus measured area fraction of connective tissue demonstrated a strong correlation between the two (*r*^2^ = 0.89, *p* < 0.001; Figure 5, dashed yellow line). The slope of the linear regression was 212, indicating that, for every 1% increase in area fraction of connective tissue, total collagen content increased by 2.12 μg/mg wet weight which is a very large number compared to muscle collagen content values measured (Figure 2). Additionally, this slope also predicts that when the area fraction of connective tissue is 100%, pure connective tissue, the collagen content of the structure is 212 μg/mg wet weight. The regression intercept of 2.81 predicts that baseline muscle collagen content is 2.81 μg/mg. Importantly, these predicted collagen content values fall within one standard deviation of the average experimentally determined dense connective tissue and baseline muscle collagen content respectively (Table 1) providing strong support for this interpretation.

**TABLE 1:** Average collagen content measured biochemically and collagen content predicted via linear regression and non-linear optimization for connective tissue structures (Col*_*ct*_*) and baseline muscle (Col*_*m*_*) in μg/mg wet weight. See text for details.

	Measured	Linear regression	Optimization
Col*_*ct*_*	276.85 ± 70.12 SD	212.43	204.45
Col*_*m*_*	2.72 ± 0.98 SD	2.81	2.80

Using the experimentally measured dense connective tissue and baseline muscle collagen content (Table 1) a predictive model (Eq. 2) derived from the law of mixtures predicts the range of total collagen content values as a function of connective tissue volume fraction (Figure 5 shaded gray region). All but one of the data points used above in the regression analysis fall within this predicted range of total collagen content values (Figure 5).

Finally, the actual values for the dense connective tissue and baseline muscle collagen contents were independently predicted using non-linear optimization (Eq. 4). The optimized values obtained of 204.45 and 2.80 μg/mg were very close to the values predicted by linear regression and similar to experimentally determined values (Table 1). Using these optimized values of dense connective tissue and baseline muscle collagen content in the previously defined the law of mixtures model (Eq. 2), the predicted relationship between total collagen content and volume fraction falls along that of the linear regression model (Figure 5 yellow dashed line) within the range observed of summed area fraction. While the regression and optimized models appear identical, they do diverge, but only at very high summed area fraction values (Supplementary Figure 3).

## Discussion

The purpose of this study was to determine how the complex connective tissue distribution within muscle affects measured collagen content. Generally, both collagen content and connective tissue structures are highest at the proximal and distal ends of the muscles (Figures 2, 4). Additionally, using three independent approaches, we found complementary and corroborating evidence demonstrating a strong relationship between muscle connective tissue structures and collagen content (Figure 5).

These results demonstrate that a single collagen content measurement typically will not accurately represent the distribution of a muscle’s connective tissue. Due to the extremely high collagen content of connective tissue, a muscle’s collagen content is driven by its major connective tissue structures and is *extremely sensitive* to the presence of even a small amount of connective tissue. As demonstrated in our analyses, for every absolute 1% increase in connective tissue volume fraction, collagen content is predicted to increase by approximately 2.12 μg/mg. These are substantial changes when compared to baseline muscle collagen content that ranges from only 1.93–4.03 μg/mg. Consequently, variations in collagen content throughout a muscle will directly reflect the internal connective tissue structures.

Previous reports of poor correlations between muscle collagen content and muscle passive stiffness are likely the result of not considering the complex variations in connective tissue structures throughout a muscle or muscle biopsy. These previous reports all compared a single collagen content value with muscle bundle or whole muscle stiffness (Smith et al., 2011; Lieber and Ward, 2013; Chapman et al., 2014; Smith and Barton, 2014). As we demonstrated, because a single collagen content value does not represent the complex connective tissue distribution throughout a muscle it is unlikely to be a good predicter of a muscle’s mechanical properties.

The methods developed above could be adapted to the *in vivo* study of collagen content and connective tissue distribution throughout a muscle using high resolution magnetic resonance imaging (MRI) (Supplementary Figure 4). Detailed biochemical and histological measurements are difficult and these laborious tasks are not feasible *in vivo*. Using the rule of mixtures model (Eq. 2) and connective tissue area fraction, as determined via high resolution MRI, collagen content throughout a muscle can be determined *in vivo*. Additionally, quantification and 3D reconstruction of connective tissues *in vivo* could be used to create physiologically realistic muscle specific biomechanical models of tissue loading that accompany mechanical data in those muscles, providing for a greater understanding of how these structures contribute to passive muscle mechanics. In order to improve the accuracy of these model predictions and account for muscle collagen content variations between species, muscles, and changes following muscle injury or diseased state, muscle and connective tissue biopsies could be taken within the muscle of interest.

### Limitations of This Study

Within this study only a select number of muscles were analyzed. However, we believe that these results are generalizable because we deliberately selected muscles that had high variations in connective tissue structures and physiological function.

Another potential limitation is that only total collagen content was measured. Connective tissue is made of multiple types of collagen with varying crosslinks, both of which may affect mechanical properties. However, the purpose of this study was to determine how total collagen content quantity and connective tissue structures relate. Future studies could perform a more detailed analysis of collagen types and crosslinks with regards to location and potential impact on mechanical properties within muscle tissue. Since type I collagen represents over 70% of muscle collagen, it is highly likely that these results reflect type I collagen throughout muscle.

Finally, a major limitation in the implications of this work is that we did not measure passive muscle mechanics. By not measuring mechanics directly, our ability to infer the impact of collagen content variations and connective tissue distribution on a muscle’s passive mechanics is speculative. However, the objective of this study was to establish methods that identify the connective tissue structures potentially contributing to a muscle’s passive mechanical properties. Future studies will use these established methods with measured passive mechanics to determine how connective tissue distribution throughout a muscle impacts passive muscle mechanics, providing a better understanding of this important function.

### Conclusions

This study demonstrated that collagen content varies widely within and between muscles and these variations are driven by the complex connective tissue’s distribution within muscle. A consequence of these findings suggests that a single collagen content measurement does not accurately represent the distribution of connective tissue throughout a muscle. Future studies should use data of collagen content variations and connective tissue anatomy to establish more accurate relationships with whole muscle passive mechanics.

## Data Availability Statement

The raw data supporting the conclusions of this article will be made available by the authors, without undue reservation, to any qualified researcher.

## Ethics Statement

The animal study was reviewed and approved by the Northwestern University’s Institutional Animal Care and Use Committee.

## Author Contributions

BB-M and RL contributed conception and design of the study, and wrote the first draft of the manuscript. BB-M and NB contributed experimental procedures. BB-M performed the statistical analysis. BB-M, NB, and RL wrote sections of the manuscript. All authors contributed to manuscript revision, read and approved the submitted version.

## Conflict of Interest

The authors declare that the research was conducted in the absence of any commercial or financial relationships that could be construed as a potential conflict of interest.
