# Supplementary material for: Intramuscular Anatomy Drives Collagen Content Variation Within and Between Muscles
Source: Front Physiol. 2020 Apr 17;11:293. doi: 10.3389/fphys.2020.00293 (PMC7181957; doi:10.3389/fphys.2020.00293)
Supplement: Supplementary file 1 [file Data_Sheet_1.PDF]

Supplementary Material

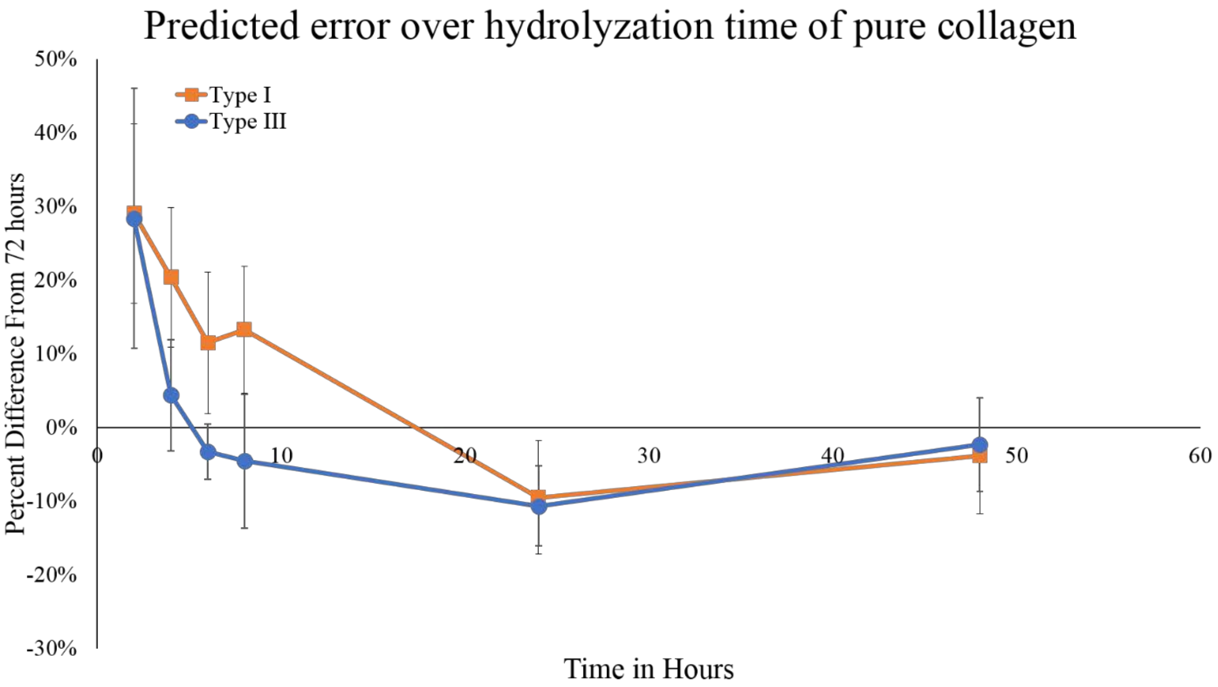

Supplemental Figure 1: Average percent difference value measured at 72 hours ± standard deviations of measured hydroxyproline content overtime of pure collagen type I (n=3 concentrations) and type III (n=3 concentrations). The hydroxyproline content peaks at 24 hours and the error associated with type I and III collagen also becomes uniform at 24 hours.

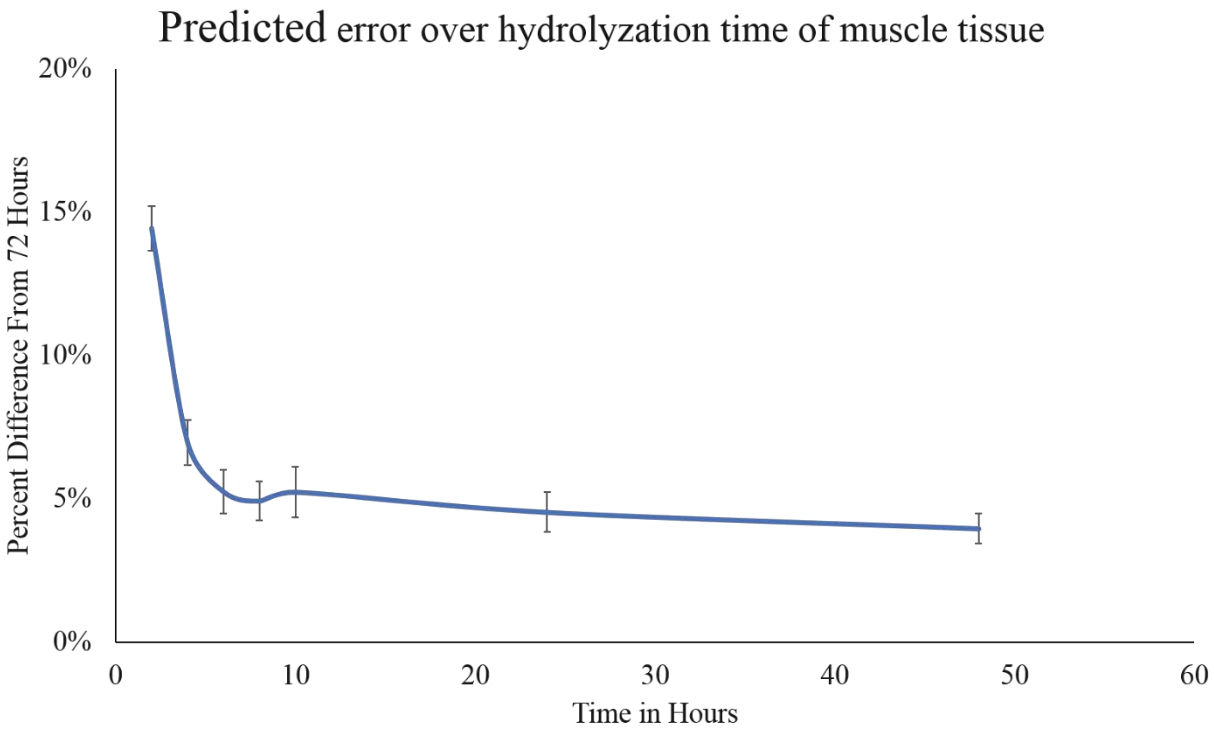

Supplemental Figure 2: Average percent difference from measured values at 72 hours ± standard deviations of measured hydroxyproline content over time of muscle samples (n=72). The predicted error as compared with the hydroxyproline content at 72 hours quickly decreases from 2 to 8 hours then levels off becoming less than 5% at 24 hours.

Analysis of contribution of loose connective tissue to total collagen content.

We conducted an additional analysis to determine the contribution of loose connective tissue to changes in collagen content within our models. As in the manuscript, we took two distinct approaches to the analysis. The first approach uses linear regression to determine how the dense (internal tendons and aponeuroses) and loose (perimysium and connective tissue surrounding neurovascular tracts) connective tissue structures summed area fraction, simplified to volume fraction below, contribute to total collagen content measured biochemically. The second uses a non-linear optimization conducting a sensitivity analysis of how loose connective tissue density (an unknown, whereas dense connective tissue density can be taken from the literature) affects the collagen content of each of the structures. In each of the analyses below the loose connective tissue's "collagen content" is not a substantial contributor to total collagen content. The collagen content of loose connective tissue from this analysis is 1000-10000 times smaller than that of the muscle tissue, contributing a negligible amount to the total collagen content. The inclusion of this factor does not improve the fit of either analysis, indicating the factor is repetitive, not adding additional information to the models, and its contribution is likely already included within the muscle tissue collagen content.

Linear Regression

Repeating the linear regression analysis done within the manuscript, the simple equation including only the dense connective tissue, the regression analysis returns:

$$Col_{tot} = Col_{dct}V_{dct} + Col_m$$

$$Col_{tot} = [212.43]V_{ct} + 2.81 \qquad R^2 = 0.89$$

Now expanding to include the "loose" connective tissue (enlarged areas of internal connective tissue structures):

$$Col_{tot} = Col_{dct}V_{dct} + Col_{lct}V_{lct} + Col_m$$

$$Col_{tot} = [212.46]V_{dct} + [0.0005]V_{lct} + 2.81 \qquad R^2 = 0.89$$

Sensitivity analysis using non-linear optimization

Repeating the optimization within the manuscript, including only the dense connective tissue and the muscle tissue, here, ρ_{dct} = 1.12 g/cm^3 and ρ_m = 1.06 g/cm^3:

$$\min_{Col_{dct}, Col_m} \left[Col_{tot}^{M,r} - \frac{Col_{dct,p}V_{dct}\rho_{dct} + Col_{m,p}V_m\rho_m}{V_{dct}\rho_{dct} + V_m\rho_m} \right]$$

$Coldc_t$	Col_m	R^2
204.45 µg/mg	2.81 µg/mg	0.89

Using the same optimization but adding "loose" connective tissue (enlarged areas of internal connective tissue structures). We can conduct a sensitivity analysis on how "loose" connective tissue

density, because there is no literature value for this structure, may affect the determination of each structure's collagen content. Where, $\rho_{dct} = 1.12$ g/cm^3 and $\rho_m = 1.06$ g/cm^3 and ρ_{lct} is varied from 0.1 to 1.2 g/cm^3 we solve the optimization below:

$$\min_{Col_{dct},Col_{lct},Col_{m,p}} \left[Col_{tot}^{M,r} - \frac{Col_{dct,p}V_{dct}\rho_{dct} + Col_{lct,p}V_{lct}\rho_{lct} + Col_{m,p}V_m\rho_m}{V_{dct}\rho_{dct} + V_{lct}\rho_{lct} + V_m\rho_m} \right]$$

ρ_{lct}	*Dense CT*	*Loose CT*	*Muscle Tissue*	R^2
0.1	203.33	0.000305	2.81	0.89
0.2	203.44	0.000615	2.81	0.89
0.3	203.55	0.001996	2.81	0.89
0.4	203.65	0.001897	2.82	0.89
0.5	203.76	0.000163	2.82	0.89
0.6	203.87	0.002614	2.82	0.89
0.7	203.98	0.003092	2.82	0.89
0.8	204.09	0.006517	2.82	0.89
0.9	204.19	0.000387	2.82	0.89
1	204.30	0.000863	2.83	0.89
1.1	204.41	0.000634	2.83	0.89
1.2	204.52	0.000843	2.83	0.89

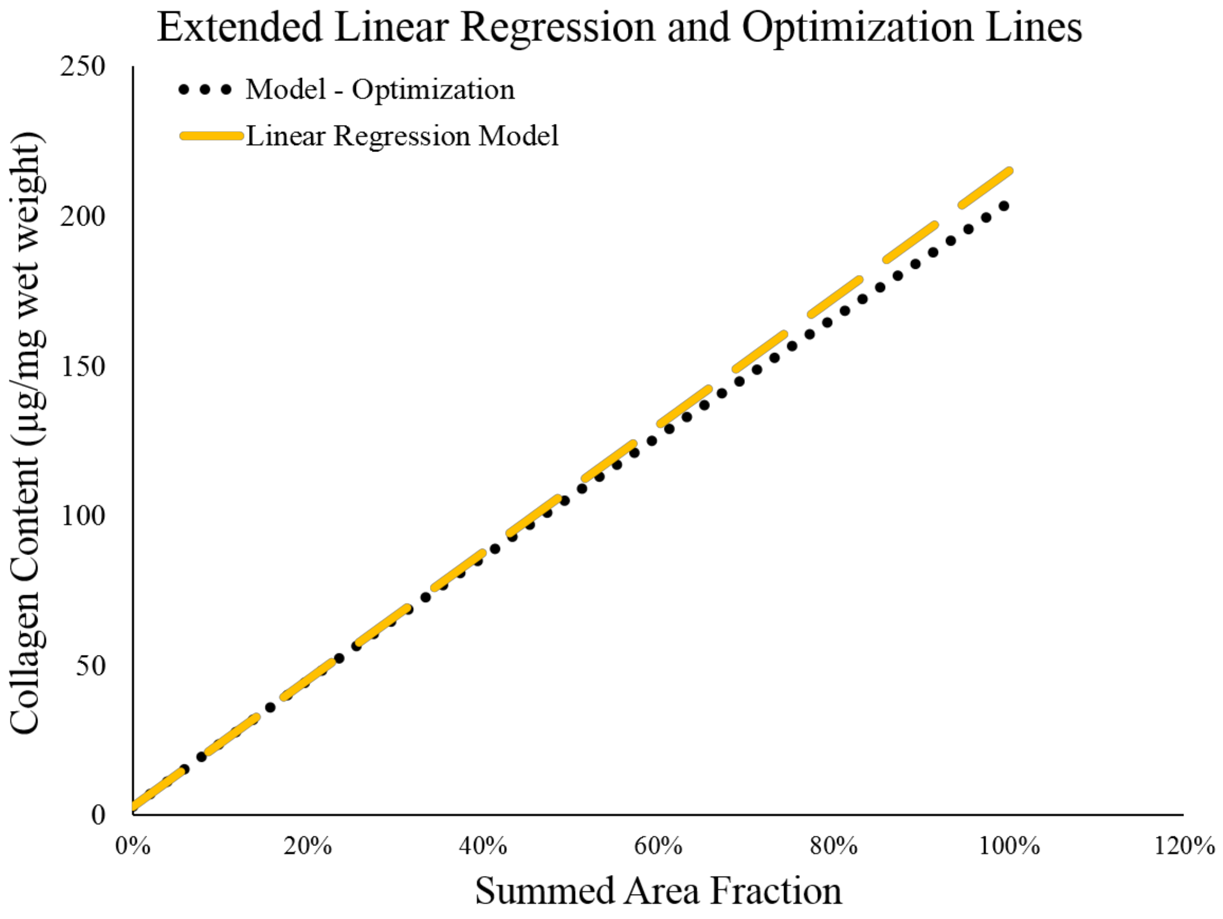

Supplemental Figure 3: Predicted relationship between collagen content and summed area fraction from the linear regression model (yellow dashed line) and law of mixtures model using the optimized predicted values of connective tissue and muscle (black dotted line) taken from 0% to 100% summed area fraction. This demonstrates the divergence of the two but only at higher area fractions and the non-linear nature of the law of mixtures model used in the optimization.

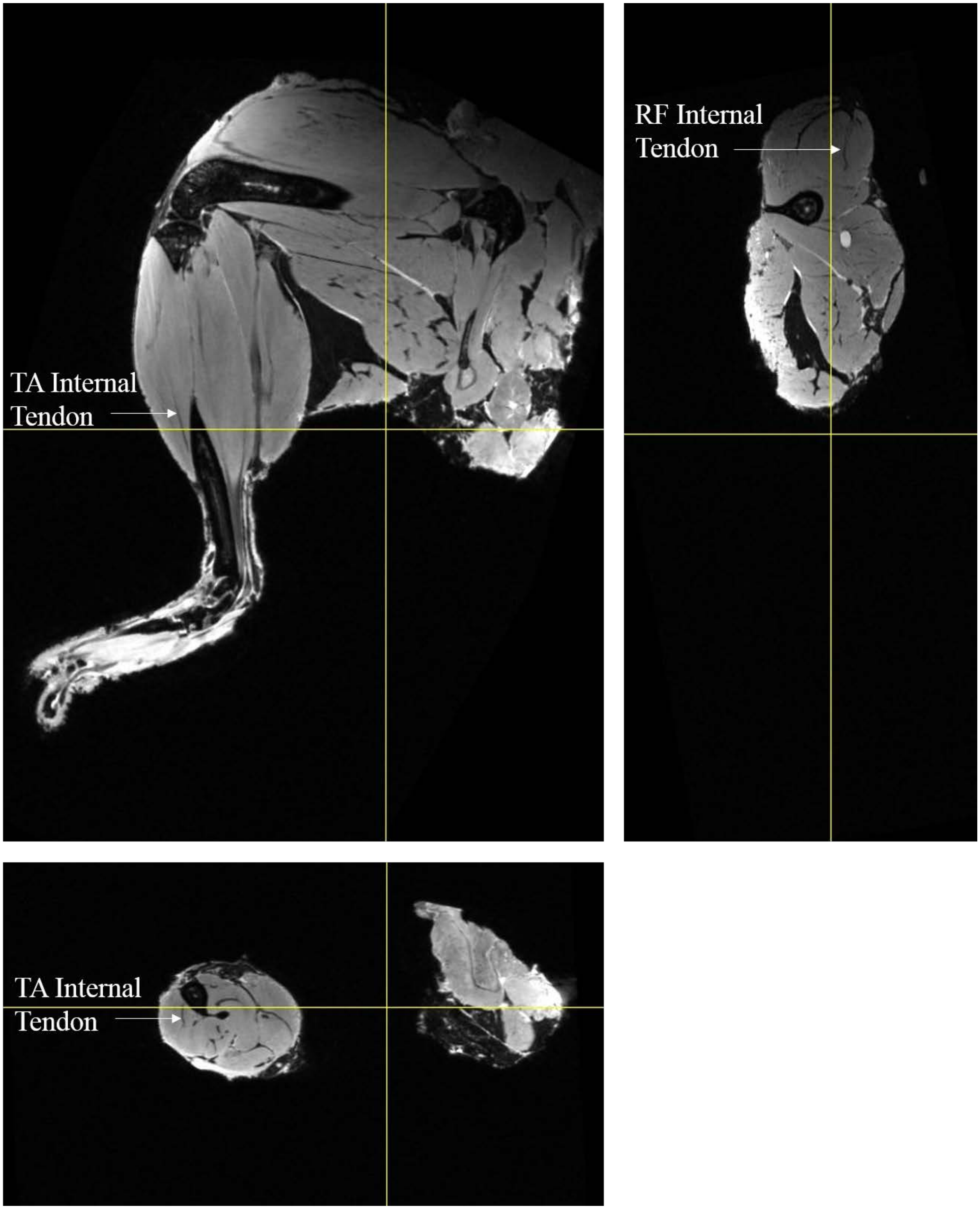

Supplemental Figure 4: Example of a high-resolution MRI of a mouse hindlimb with 50μm voxel resolution with the internal tendons of the tibialis anterior (TA) and rectus femoris (RF) identified. Other muscles and connective tissue structures are visible and could be analyzed by modifying the methods described in the paper. Yellow lines denote planes at which orthogonal images were taken.

Supplemental Table 1: Average ±SEM measured baseline muscle collagen content of the rectus femoris (RF), semimembranosus (SM), tibialis anterior (TA), and lateral gastrocnemius (LG) in μg/mg wet weight (n=6/muscle).

	Collagen content
RF	1.93 ±0.07
TA	4.03 ±0.42
LG	2.34 ±0.15
SM	2.56 ±0.17

Supplemental Table 2: Average ±SEM measured connective tissue collagen content of the internal tendons of the rectus femoris (RF-IT; n=6) and tibialis anterior (TA-IT; n=6) and aponeurosis of the lateral gastrocnemius (LG-AP; n=6) and rectus femoris (RF-AP; n=3) in μg/mg wet weight.

	Collagen content
RF-IT	258.84 ±39.44
TA-IT	261.70 ±24.44
LG-AP	334.69 ±7.15
RF-AP	227.53 ±16.00
